# Running Away to the Circus

**Published:** 2007-12-15

**Authors:** Lynne S. Wilcox

The novel, *Water for Elephants* (1), opens with a circus hand who abruptly finds himself in the midst of a menagerie stampede. He fights his way through yaks, hyenas, bears, chimpanzees, and terrified customers into the Big Top to rescue a young woman and an elephant. This exotic opening begins the story of a young man — the circus hand — who walks out of his veterinary final examinations and, through a series of unexpected events, takes up with a traveling circus during the Great Depression.

We soon discover these are the memories of Jacob Jankowski, who is "[n]inety. Or ninety-three. One or the other." He doesn't mind so much that he can't remember his age, and he's grateful that his hip surgery allows him to get about with a walker (although he also uses a wheelchair). But Jacob is in "the old folks' home." Sitting down to mushy peas in the dining room, when he still has teeth and wants pot roast and corn on the cob, affronts his dignity. It only gets worse when he starts an argument with another resident at dinner and is sent to his room like a misbehaving child.

Mr. Jankowski is a member of the population group called the oldest old. This issue of *Preventing Chronic Disease* discusses the health concerns of these seniors. We thank Dr. Jaya Rao of the Division of Adult and Community Health, National Center for Chronic Disease Prevention and Health Promotion, Centers for Disease Control and Prevention, for serving as guest editor for this issue.

From 2000 to 2030, the number of people aged 80 years or older in the United States will double to 19.5 million (2). Demographers have predicted this outcome since the baby boomer generation was recognized, and we now have a list of survival instructions for the oldest old. Optimal preparation for the ninth decade and beyond includes physical activity, proper nutrition, tobacco avoidance, social networks, cognitive challenges, and available health care.

However, quality of life in the last years depends on more than the items in this list; it requires *resilience*. Lavretsky and Irwin's review of the literature on resilience defines it as the ability to maintain biologic and psychologic homeostasis under stress (3). Stress can range from severe illness to loss of a loved one to life in wartime. Researchers from the Notre Dame Study of Optimal Aging found the frequency of positive emotions and the ability to recognize complex emotions were associated with an older person's ability to withstand stress (4,5). A study of older, chronically ill African Americans found that attitudes of independence, spirituality, and survival defined resilience (6). The German Heidelberg Centenarian Study identified resilience traits of self-efficacy and optimism (7). A Swedish study of elders reported equanimity, meaningfulness, perseverance, existential aloneness (the ability to enjoy being alone without feeling lonely), and self-reliance as key factors in resilience (8).

Is resilience inborn, or does it develop over time? Can historical circumstances create resilience? Howe and Strauss popularized the concept that different U.S. generations hold different world views, depending on their life experiences (9). They refer to people aged 85 through 100 years on January 1, 2007, as the GI generation. This cohort experienced and is affected by the Depression, the New Deal, and World War II. Major generational events of baby boomers (the group born from 1943 through 1964) included the Cold War; the assassinations of John and Robert Kennedy and Martin Luther King, Jr; the Vietnam War; and the civil rights movement.

Can resilience be actively promoted? Will resilience vary between the GI and boomer generations as their histories vary? We know aging experiences will differ; for example, the baby boomer cohort will see more of its members survive to the oldest ages, and those seniors will be healthier. These differences could affect the health advice we offer to the oldest old. We will need to discover what characteristics remain constant across generations and what characteristics history may affect.

Meanwhile, what of Jacob Jankowski? Throughout the story, we learn of his present and his past (1). He spent a lifetime working for the circus. He married and had children, then settled down. Now he is as physically active as possible. He receives balanced nutrition, housing, health care, and cognitive stimulation in the retirement facility. He doesn't smoke. Although he is a widower, he has an extended family that visits him every weekend. But is he *resilient*?

Today the circus is in town, just down the street from the old folks' home. One of Jacob's sons is scheduled to take him there. Jacob waits in the lobby in his wheelchair, and his son doesn't appear. One by one, the families of the other residents arrive, but still Jacob waits, impatient to see the Big Top one last time. Then he learns his son has forgotten their appointment, on today of all days. It's not fair, Jacob thinks. He's stuck in this lobby.

Or is he?

Carefully Jacob transitions from his wheelchair to his walker. Step by shuffling step, he moves out of the lobby, though the automatic doors, onto the sidewalk. Then he heads down the street, 10 inches at a time, making steady progress. Jacob Jankowski, age 90 or 93, one or the other, is running away to the circus.
